# Possible Therapeutic Use of Spermatogonial Stem Cells in the Treatment of Male Infertility: A Brief Overview

**DOI:** 10.1100/2012/374151

**Published:** 2012-03-12

**Authors:** Slobodan Vlajković, Rade Čukuranović, Marija Daković Bjelaković, Vladisav Stefanović

**Affiliations:** Faculty of Medicine, University of Niš, 18000 Niš, Serbia

## Abstract

Development of germ cells is a process starting in fetus and completed only in puberty. Spermatogonial stem cells maintain spermatogenesis throughout the reproductive life of mammals. They are undifferentiated cells defined by their ability to both self-renew and differentiate into mature spermatozoa. This self-renewal and differentiation in turn is tightly regulated by a combination of intrinsic gene expression as well as the extrinsic gene signals from the local tissue microenvironment. The human testis is prone to damage, either for therapeutic reasons or because of toxic agents from the environment. For preservation of fertility, patients who will undergo radiotherapy and/or chemotherapy have an attractive possibility to keep in store and afterwards make a transfer of spermatogonial stem cells. Germ cell transplantation is not yet ready for the human fertility clinic, but it may be reasonable for young cancer patients, with no other options to preserve their fertility. Whereas this technique has become an important research tool in rodents, a clinical application must still be regarded as experimental, and many aspects of the procedure need to be optimized prior to a clinical application in men. In future, a range of options for the preservation of male fertility will get a new significance.

## 1. Introduction

In the normal human testis there are few cells, which are important for such a multistaged process as spermatogenesis. The most important cells are germ cells in their various developmental stages, supporting Sertoli cells in the seminiferous tubules, and interstitial Leydig cells producing hormone testosterone, which is necessary for normal process of spermatogenesis.

 Normally, primordial germ cells differentiate into gonocytes, which transform to fetal spermatogonia from 10 to 22 weeks post conception. Fetal spermatogonia begin to transform into adult dark (A_dark_) spermatogonia. Diploid spermatogonial stem cells (SSCs) or type A_dark_ spermatogonia have characteristic adult stem cell properties of self-renewal and differentiation. Through assymetric cell division, they replace themselves and produce more differentiated progenitor daughter cells, also known as adult pale (A_pale_) spermatogonia. Although both (A_dark_ and A_pale_) are commonly referred to as spermatogonial stem cells, their biological functions are very different and the A_dark_ shows characteristics indicating that it acts as a testicular stem cells. The progeny of A_pale_ are B spermatogonia. They proliferate and differentiate to form four spermatocytes. Meiosis ensues to produce haploid spermatids. It takes about 64 days after a single SSC division, which gives rise to 16 haploid spermatids before mature spermatozoa are formed [1]. The spermatozoa are released into the lumen of seminiferous tubules and are transported to the epididymis where they continue to mature. Final steps of spermatogenesis occur at puberty. During this period, the Sertoli cells develope, and their total number decreases constantly from birth to puberty. The mutual interaction between germ cells and Sertoli cells plays a crucial role in their differentiation. The cytokines produced by Sertoli cells regulate spermatogonial and spermatocyte development, junctional integrity, and the function of immunoregulatory cells present in interstitium [2]. Leydig cells degenerate to minimal numbers by the age of two years. At puberty, they differentiate to adult Leydig cells [3]. Peritubular myoid cells surround the seminiferous tubules and express androgen receptors from fetal life to adulthood. Recently, the molecular mechanisms of androgen action via these cells in spermatogenesis have been identified, which is essential for normal testis function [4], but, as demonstrated by O'Shaughnessy and colleagues, androgen stimulation of spermatogenesis, nevertheless, requires direct androgen action on the Sertoli cells [5]. Identifying Colony stimulating factor 1 (Csf1) as an extrinsic stimulator of SSC self-renewal, it was implied that Leydig and peritubular myoid cells are contributors of the testicular stem cell niche in mammals [6]. As a plasma membrane component, among other glycolipids in the mammalian testis, testis-specific sulfoglycolipid, seminolipid, is essential for germ cell function in spermatogenesis [7].

 Induction of spermatogenesis depends on the complementary actions of follicle-stimulating hormone (FSH) and androgens. FSH is capable to establish a sufficient Sertoli cell population, while androgens (mostly testosterone) affect the functional completion of meiosis and postmeiotic sperm differentiation and maturation. Luteinizing hormone (LH) stimulates Leydig cell production of testosterone. FSH alone can induce proliferation of Sertoli cells and spermatogonia in the prepubertal primate, but this does not result in qualitatively and quantitatively normal spermatogenesis unless testosterone is simultaneously present [8, 9]. Although FSH appears to play a more prominent role in the maintenance of primate spermatogenesis than in the initiation, normal spermatogenesis is best maintained by the combined effects of FSH and LH [8]. As Plant and Marshall claimed, FSH stimulation is probably not obligatory for the maintenance of spermatogenesis, but it is premature to infer that LH is sufficient and necessary [10]. Recently, Achard and colleagues showed that complete and quantitatively normal spermatogenesis may be triggerred and maintained by low levels of luteinizing hormone activity postnatally and at puberty [11].

**Figure 1:**
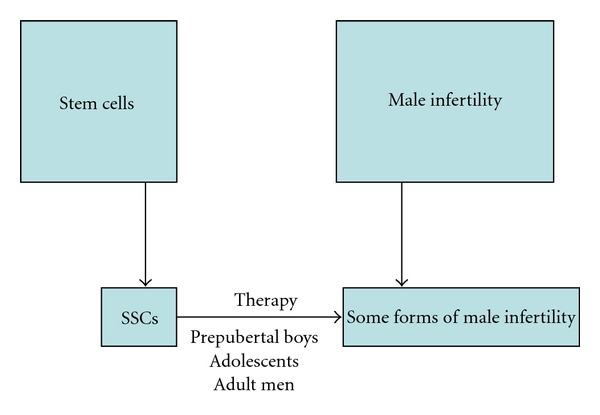
Possible therapeutic application of spermatogonial stem cells (SSCs).

 Stem cells are reserve cells capable of indefinite self-renewal. They have an extraordinary potential for therapeutic use in regenerative medicine. Stem cells are controlled by particular microenvironments known as niches. Male germline stem cells, SSCs, have the capacity to continuosly produce sperm during adult life, and they do it through establishment of an SSCs population during early testis development and its subsequent maintenance [12]. In recent years, a few strategies have been introduced for preserving fertility in prepubertal boys, adolescents, and adult men, where it is necessary to apply anticancer therapy, or where the infertility in man occurred for some other reasons. All of these strategies include the application of SSCs in the treatment of infertility. For these reasons, the aim of this review was to make the connection between the SSCs and main causes of male infertility, in which therapy they could find application, which refers to the treatment of men of different ages—prepubertal boys, adolescents, and adult men (Figure 1). This is done through a review of contemporary knowledge about these cells and some causes of male infertility.

## 2. Male Infertility and Causes

Infertility can be caused by defects in the development of the urogenital system and in its function, by genetic defects of the endocrine system, and by defects in gametogenesis, erection, ejaculation, gamete function, fertilization, or early embryonic development. Secondary or acquired infertility can occur, among other causes, due to exposure to gonadotoxins [13]. The most common causes of male infertility include abnormal sperm production or function, impaired deliver of sperm, and overexposure to certain gonadotoxins from the environment. The pathogenesis of male infertility can be attributed to the disorder of germ cell proliferation and differentiation or to somatic cell dysfunction [14]. Among other causes, cryptorchid testes and testicular cancer play an important role, as well as influence of radiotherapy and/or chemotherapy used in the (other) cancer treatment. Animal models, particularly knockout mouse models, and their various applications are of great importance in studies of male infertility [15].

 Cryptorchidism is a common condition, with significant risks of infertility and malignancy. The mechanism of normal testicular descent from an intraabdominal position to an extraabdominal position (scrotum) occurs in two basic steps. The first step is transabdominal phase, when testis is held close to the inguinal region. This phase is triggered by hormonally controlled enlargement of the distal gubernaculum. During the second, the inguinoscrotal phase, the gubernaculum migrates across the pubic region into the scrotum. This phase is indirectly controlled by androgens via the genitofemoral nerve and release of neurotransmitters, especially calcitonin gene-related peptide (CGRP) [16]. There are two forms of cryptorchidism: congenital (cryptorchidism A) and acquired (cryptorchidism B). Congenital form is caused by any abnormality of the anatomical or hormonal mechanisms. Unilateral undescended testis occurs because androgens act independently on each side via the ipsilateral genitofemoral nerve [17]. Acquired form is caused by failure of postnatal elongation of the spermatic cord, which, normally, should be doubled in length by the 10th year [18]. The scrotal testis is cooler than in the body cavity and is programmed to function at lower temperature after birth. Hormon production and germ cell development fail when the testis is located in the body cavity, which leads to subsequent infertility and increased risk of malignancy. Transformation from gonocyte to type A spermatogonium is inhibited in these conditions [19]. But, when the cryptorchid testis is returned to the scrotum, complete spermatogenesis is restored indicating that SSCs remain functional [20]. Because differentiated germ cells are absent in the cryptorchid testis, this testis cell population might be expected to contain a greater concentration of stem cells. Shinohara and colleagues showed in mouse models that the total number of SSCs is approximately the same in wild-type and cryptorchid testes, indicating that the elevated temperature had little or no effect on stem cells [21].

 The human testis is an organ known for damage caused by exposure to therapeutic agents and harmful factors from the environment [22]. It has very active systems that work together to regulate the extent of germ cell apoptosis. Germ cells excessively proliferate, and physiological apoptosis optimizes their output to a satisfactory level for the spermatogenesis. Uneven apoptosis can result in decreased sperm output. After the exposure to a different testicular toxins, apoptosis significantly increases and leads to germ cell damage and/or seminiferous epithelium becomes dysfunctional [22]. Various agents are harmful to spermatogenesis in different animal models, and some of them are selective for germ cells, some for Sertoli cells, while some affect the Leydig cells. In this way, spermatogenesis is suppressed either by damage of germ cells, or by impossibility to create microenvironment by Sertoli cells, or by lack of testosterone.

 Chemotherapy with different agents, like cysplatin, could have profound effects on spermatogenesis and various consecutive damages. In adult rats, there is a significant reduction of testosterone in serum and testes seven days after exposure to cisplatin. The germ cells die rapidly due to apoptosis induced by cisplatin [23]. Spermatocytes are more sensitive than spermatogonia to the effects of cisplatin. It causes long-lasting azoospermia and testicular atrophy in men. Therefore, it is likely that cisplatin targets multiple cell types and molecular pathways while producing testicular injury [22].

## 3. Stem Cells Characteristics

Three main characteristics define stem cells: self-renew ability, the ability to differentiate into one or more lineages of specialized cell types, and an enormous proliferative potential for the maintenance of the tissues they populate [24, 25].

 The physiological role of a stem cell includes coordinated control of growth and differentiation, as well as induction of apoptosis, which distinguish them from malignant cancer cells [26]. Stem cells allow blood, bone, gametes, epithelia, nervous system, muscle, and many other tissues to be replenished by fresh cells throughout life [27]. In most systems, the stem cells do not derive finally differentiated cells directly but do so through progenitor cells. These progenitors are intermediate cell populations inserted between stem and differentiated cells. Thus, the stem cells play the role of a regenerative reserve, and progenitor cells play the role of a functional reserve, producing exactly the number of differentiating cells needed for tissue homeostasis [1].

 Stem cells are classified according to their developmental potential as totipotent, pluripotent, multipotent, and unipotent. A totipotent stem cell, zygote and its offspring cells of morula, can give rise to a new individual. A pluripotent stem cell can give rise to all cell types of the embryo proper, including somatic and germ cells. Adult stem cells are multipotent if they are able to differentiate into multiple cell types of a single tissue. Examples include haematopoietic stem cells, mesenchymal stem cells, and neural stem cells. They are isolated from the developing germ layers and/or its descended adult organs [28]. The unipotent cells, or precursor cells, exhibit limited or no capacity for self-renewal and are able to contribute only to one mature cell type.

 Adult stem cells were first described in tissues characterized by a high rate of cell turnover, such as blood, skin, gut, and testis. Recent reports claim that stem cells can be even found in most adult organs that do not display high rate of cell turnover [25]. Stem cells are controlled within restricted tissue microenvironments known as “niches”. A niche consists of a local tissue microenvironment capable of housing and maintaining one or more stem cells. It is localized and not a general tissue property. Niches integrate local and systemic signals for the regulation and maintenance for resident stem cells [27]. Two basic types of niche have been recognized at the tips of adult Drosophila female and male gonads. “Stromal cell” niches develop whether or not stem cells are present and maintain their morphology after stem cell loss. They have distinct “stromal” cell types—cap cells and hub cells which are in direct contact and signal to resident stem cells. The stromal cells often secrete growth factors to regulate stem cell behavior [29]. Stem cell niches have a different configuration in various tissues. In addition to maintaining stem cells during division, they must prevent external cells from gaining entry and displacing the resident stem cells. The existence of a mechanism for stem cell replacement might also have deleterious effects over the course of a long lifespan in tissue with a large number of stem cells and niches. Mutations within stem cells, that enhance the ability of daughter cells to target and replace nearby stem cells, would tend to increase their representation among the stem cells within a tissue [30]. Also, the data from Drosophila germ line stem cells show a phenomenon, which might also exist in mammals, where two different types of stem cells share the same niche [31]. Singh and colleagues concluded that Janus kinase/signal transducer and activator of transcription (JAK/STAT) signaling controls competitiveness for the niche and mutual dependence of SSCs and somatic cyst progenitor cells [32]. Finally, it was established that pluripotent stem cells can be directly generated from fibroblast cultures (reprogramming) by the addition of only a few defined factors. This finding may eventually allow the creation of pluripotent cells directly from somatic cells of patients [33–35].

## 4. Spermatogonial Stem Cells

Spermatogonial stem cells (SSCs) are specific germ cells that differentiate to initiate the process leading to the formation of sperm [32]. They are undifferentiated cells defined by their ability to both self-renew and differentiate into mature spermatozoa. Although critically important for the production of sperm, SSCs have been difficult to study because of their small number in the testis and challenges associated with identifying, culturing, and assaying their biological activity [36]. They may differentiate into various types of somatic cells under specific conditions *in vitro *and form teratomas after inoculation into mice [37].

 SSCs or testicular stem cells originate from primordial germ cells that travel to the gonadal ridges during embryonic development. After migration into the undifferentiated gonads, the primordial germ cells differentiate into female or male germ cell precursor depending on the sexual gonadal differentiation [38]. After the prepubertal initiation of germ cell differentiation, spermatogenesis is maintained by the ability of SSCs to provide a continual supply of differentiating spermatogonia. SSCs have an ability to self-renew, to create additional stem cells and cells destined for differentiation. To maintain this ability, like other adult stem cells, SSCs need to reside in a unique environment, or niche, that provides the factors for their survival and potential. Ehmcke and colleagues concluded that a variety of different totipotent and germ line cells are capable to enter meiosis under *in vitro* conditions but that male germ cell differentiation occurs exclusively in the intact testicular microenvironment [39]. They claim, also, that the testicular microenvironment offers unique niches to germ cells. Physically, the SSC niche most likely lies along the basement membrane of the seminiferous tubule, with the Sertoli cells contributing to this microenvironment [36]. The Sertoli cells are specialized cells which provide the nutritional and architectural support required for adult germ cell development [12]. In mammals, the somatic Sertoli cell is responsible for maintaining the SSC. Sertoli cells create formation of SSC niches through secretion of specific growth factors, and inducing output of secreted factors from Leydig cells and other interstitial cell populations [40]. Until recently, it was suggested that a single Sertoli cell factor, glial cell line-derived neurotropic factor (GDNF), which is a protein member of the TGF-*β* superfamily, is most likely responsible for that. Now, there are data suggesting that SSC regulation changes as the testis develops from perinatal to pubertal age, the perinatal period is regulated by GDNF, and the pubertal period is dependent on Ets-related molecule (ERM). ERM is localized in the Sertoli cell, the only somatic cell of the seminiferous epithelium, and it was established that in adult testes Sertoli cells maintain the SSC niche. This molecule is essential for stem cell renewal in pubertal and adult testes [41]. The number of SSCs increases from birth to sexual maturity, when the seminiferous tubule appears to provide an environment supportive of the formation of new niches. It was suggested that SSCs can develop new niches during the initiation of spermatogenesis [34]. Also, there is an evidence that SSCs exhibit different phenotypes in different biological microenvironments [42].

 In the testis, SSCs, residing in a niche, can regenerate spermatogenesis even following a toxic insult [43]. In contrast, damage to the niche or Sertoli cell microenvironment may limit or prevent SSC engraftment [12]. There is also a significant opportunity that the embryonic stem cells are able *in vitro* to give rise to SSCs that can produce functional gametes which are able to fertilize oocytes [44].

 Nowadays, there are two crucial questions for a more complete elucidation of the role of SSCs: what is the signal that stimulates an SSC to begin the process of differentiation, and what is the signal that stimulates an SSC to divide to self-renew the SSCs population [45].

## 5. Spermatogonial Stem Cells in the Treatment of Infertility

Grafting of isolated testis cells has been developed more recently, and as such it has been explored less than tissue xenografting. There is the remarkable capability of isolated postnatal testis cells to recapitulate testis development and undergo complete differentiation [46]. SSCs are unique among the adult stem cells because they are the only self-renewing population of cells that genetically contribute to the next generation [43]. Infertility after testicular exposure to moderate doses of radiation and some chemotherapeutic agents occurs as a result of inability of spermatogonia to differentiate. After cytotoxic therapies, germ cells appear to be absent, and the tubules contain only Sertoli cells. This could be a result of killing the SSCs, the loss of ability of the Sertoli cells to support the differentiation of SSCs, or both [47]. In those patients whose anticancer therapy clinically predicts a complete depletion of SSCs, the outlined approaches of germ cell transplantation and testicular grafting might offer options for fertility preservation. A technique for transplanting SSCs was first described by Brinster and colleagues [48, 49]. Restoration of fertility following SSCs transplantation in rodents suggests therapeutic potential for the technique in humans. Further research is necessary, especially in primate models, and the cryopreservation of testicular cells and/or tissue should be considered an significant aspect of oncological therapy [50]. For preservation of fertility, an attractive proposition is the storage and transfer of SSCs. The gonocytes can be frozen-stored prior to transfer and still produce fertile seminiferous tubules. When SSCs are harvested from donor testes and transplanted into a sterilized recipient testis, morphologically and functionally normal spermatogenesis is reestablished [13]. Rat gonocytes produced mature spermatozoa in testes of immunodeficient mice [51]. They were able to fertilize oocytes by *in vivo* fertilization (IVF) but with reduced fertilization and development rates in the transplanted group, where live born pups did not show anomalies, but one was observed with a lower pregnancy rate and a smaller litter size in females impregnated with transplanted male mice [52]. This difference may be due to the lower motility in the epididymal sperm of transplanted animals [53]. Also, the testis appears tolerant to foreign cells, especially because of blood-testis barrier, even the interstitium which is outside this barrier. The blood-testis barrier maintains a selective flow between luminal fluid, interstitial fluid, and plasma, creating an immune-privileged environment for germ cells [54]. The number and quality of semen specimens is often unsatisfactory, deteriorating further with cryopreservation, and it is not an option for prepubertal boys [55].

 Currently, male cancer patients, prior to receiving sterilizing doses of chemotherapy and/or radiotherapy, may be offered semen cryopreservation followed by thawing and insemination. For preservation of fertility, autographs avoid both the immunological problems of allografts and the ethical dilemma when using donor tissue. The banking of at least three semen samples with an abstinence of at least 48 hours between samples is good choice, and it has been recommended [56]. Adolescents and adult men have the option of cryobanking their semen before cancer treatment and, by IVF or intracytoplasmic sperm injection (ICSI), they can become fathers of children who are genetically their own. In contrast, prepubertal boys cannot benefit from this approach since they do not have completed spermatogenesis, because their seminiferous epithelium contains only Sertoli cells and different types of spermatogonia, among which are the SSCs [57]. But, it is possible to develop the successful transplantation of SSCs and Leydig cell progenitors, conserving the fertility of juvenile patients undergoing radiotherapy during cancer treatment [43, 58]. A necessary step in saving the fertility of young male human cancer patients will be by way of taking a biopsy before chemotherapy, propagation of SSCs in culture, cryopreservation of the cells, and transplantation back to the patients after a cure and after puberty [59–62]. Testicular biopsy and tissue cryopreservation hold promise for this young patients, but additional scientific advances are still needed to translate successes in animal research to human clinical practice, and the latest results show the attempts of scientists to achieve *in vitro* propagation of SSCs in humans [63, 64]. Preservation of testicular tissue from prepubertal patients will allow them to consider various fertility restoration options that will emerge in next two or three decades, giving them hope of fathering children with their own genetic heritage [65]. It was shown that immature testicular tissue has a surprisingly high potential to survive and differentiate as an auto- or xenograft [66].

 It has already been proven that SSCs, present in testes of patients with nonobstructive azoospermia, can be isolate and propagate *in vitro* using the highly efficient culture system and produce differentiating germ cells with developmental potential [67]. Hence, it would be taken into account that the testis biopsy taken from the cancer patient may contain malignant cells. These cells should be removed from the cell suspension because one single malignant cell may reintroduce the disease [68]. Thus, application of negative biomarkers for SSCs should allow depletion of tumor cells from a testis biopsy, which assure protection against tumor relapse [69]. Further, there is a need for the preparation of recipient, which involves the destruction of endogenous germ cells and blockade of spermatogenesis, to allow transplanted SSCs to translocate from the lumen to the basal compartment of the seminiferous tubule and begin donor-derived spermatogenesis. Based on experiments on mice, it is showed that potential safety hazards, associated with busulfan or other cytotoxic treatments, can be avoided by heat shock treatment (testicular hyperthermia), during which spermatogonial niches stay maintained and capable of supporting donor-derived spermatogenesis [70].

 If chemotherapy and/or radiotherapy have already been started, collection and cryopreservation of semen are still feasible during treatment, at least until azoospermia ensues. In these cases, the effects of these gonadotoxic agents on sperm are unknown. But, testicular sperm extraction is possible from nonobstructive azoospermic cancer patients, which in combination with ICSI gives a potential new treatment option [56]. The studies revealed that differences in the regulation of spermatogenesis do not allow the xenodifferentiation of germ cells, most likely because of disturbed communication between nonrodent germ cells and a mouse seminiferous epithelium [1]. Nowadays, it is demonstrated that the organ culture conditions can support the complete spermatogenesis of mice [71]. Also, it is not known whether the offspring, especially those produced from cryopreserved tissue, are healthy in general, but fertility of the offspring is just a crude indicator of whether gametes are “normal” or not [72].

## 6. Perspectives

Exciting research in SSCs transplantation offers the potential for future therapy to restore fertility in previously infertile men. In the future, by performing experiments, mostly on small mammals, there will be many new information on using these germ cells for possible preservation of fertility. For this purpose, spermatogonial and other types of stem cells will be used. For example, adult bone marrow cells, in a favorable testicular environment, differentiate into somatic and germ cell lineages [13]. Further, the isolation of germ line-competent cells from sources other than the testis may render it possible to even use female cell preparation as donor cell preparations for germ cell transplantation and the reinitiation of spermatogenesis [1]. Also, by using better protocols for cryopreservation and cryostorage, success of fertility preservation, via testis tissue or SSCs transplantation, will be mostly guaranteed. Finally, it should be noted that SSCs may also offer a renewable source of cells to be used to correct many diseases of aging, to develop new cell-based therapies, and to advance germline gene therapy [12].
